# Supplementary material for: Content-rich biological network constructed by mining PubMed abstracts
Source: BMC Bioinformatics. 2004 Oct 8;5:147. doi: 10.1186/1471-2105-5-147 (PMC528731; doi:10.1186/1471-2105-5-147)
Supplement: Additional File 5 — The original Chilibot query results of the term "long-term potentiation (LTP)" and 22 other terms, limiting the latest references analyzed to the years 1990, 1995, 2000, and 2004. [file 1471-2105-5-147-S5.bz2 › chilibotAdditionalFile5/ltp1995/html/ACTIN_ACTININ.html]

 


 **ACTIN** and **ACTININ** 
  
Found 648 abstracts in PubMed,  **30 abstracts were retrieved and analyzed**.  


---

 Search Google  |
 PDF files only 
|  EDU domain only 

---

**Interactive relationship** (e.g. stimulation, inhibition, etc)

- Determination of the protein components of native thin filaments isolated from natural actomyosin nebulin and alpha  **actinin**  are associated with  **actin**  filaments.  Ref: 8543580 J Biochem (Tokyo), 1995
- Despite conservation of the  **actin**  binding site, fish alpha  **actinin**  has a better  **actin**  binding ability kD = 0.3 microM than chicken smooth muscle alpha  **actinin**  kD = 1.6 microM.  Ref: 7584856 Comp Biochem Physiol B Biochem Mol Biol, 1995
- Both alpha  **actinin**  and nebulin were cosedimented with  **actin**  filaments, while connectin was not.  Ref: 8543580 J Biochem (Tokyo), 1995
- Confocal microscopy showed alpha  **actinin**  to be localized along  **actin**  stress fibers in wild type cells, and in 5.51 cells stress fibers were absent and alpha  **actinin**  was associated with F  **actin**  in the filopodia.  Ref: 7493629 Exp Cell Res, 1995
- Dystrophin, beta spectrin, alpha  **actinin** , ABP 120, ABP 280, and fimbrin share homologous  **actin**  binding domains and comprise an  **actin**  crosslinker superfamily.  Ref: 7635207 FEBS Lett, 1995
- along with the unsuitability of  **actin**  polymers from oxidized monomers to interact with both filamin and alpha  **actinin** , suggest that hydrogen peroxide influences  **actin**  dynamics mainly by changing the F  **actin**  structure.  Ref: 8599677 Biophys J, 1995
- Moreover, we have recently reported that alpha  **actinin**  and filamin display divergent behaviours towards conformational changes of  **actin** .  Ref: 8567941 J Muscle Res Cell Motil, 1995
- Northern blot analysis of mRNAs for the intracellular contractile proteins revealed that interferon alpha 2b significantly down regulated mRNA levels of the  **actin**  isoforms beta and gamma 50% to 60% but had no significant effect on alpha tubulin, vimentin, and alpha  **actinin** .  Ref: 7595033 J Lab Clin Med, 1995
- Consequently, the 550 kDa protein may play an important role in the binding of myofibrils to the basal lamina by interaction with F  **actin** , alpha  **actinin** , laminin, fibronectin or Type IV collagen.  Ref: 7492578 Biochim Biophys Acta, 1995
- Exposure of cells for 3 h to the same supernatant, prior to addition of oxytocin in the culture medium, abolished oxytocin responsiveness, had no effect on immunolocalization of  **actin**  and vimentin, but affected the localization of alpha  **actinin**  within myoepithelial cells.  Ref: 8568028 J Dairy Res, 1995

**Parallel relationship** (e.g. studied together, co-existance, homology, etc.)

- Moreover, we show that alpha  **actinin**  coimmunoprecipitates with the N cadherin catenin complex in an  **actin**  independent manner.  Ref: 7790378 J Cell Biol, 1995
- The addition of 0.2 unit mL thrombin caused aggregation dependent redistribution of cytoskeletal proteins  **actin**  binding protein, talin, vinculin, and alpha  **actinin** , glycoproteins GPIIb IIIa, PECAM, and signaling molecules PI3 kinase, pp60c src to the cytoskeletal fraction of platelets.  Ref: 7626626 Biochemistry, 1995
- Basic fibroblast and platelet derived growth factors as modulators of  **actin**  and alpha  **actinin**  in chick myocardiocytes during development.  Ref: 7675799 Proc Soc Exp Biol Med, 1995
- Although the reason for fish white muscle alpha  **actinin**  s close affinity to  **actin**  was not clearly established, our results suggested that the physicochemical environment of the Z filaments in Z disks might be crucial.  Ref: 7584856 Comp Biochem Physiol B Biochem Mol Biol, 1995
- Cytoplasmic and cytoskeletal concentrations of  **actin**  and alpha  **actinin**  were dependent on the stage of embryonic development and on the type of growth factor added to the culture.  Ref: 7675799 Proc Soc Exp Biol Med, 1995
- Using profile methods, we have detected two repeats of this domain in the  **actin**  binding region of alpha  **actinin**  and related proteins.  Ref: 7589522 FEBS Lett, 1995
- The 190 kDa fragment possessed similar levels of activities to stimulate both polymerization of G  **actin**  and alpha  **actinin**  dependent gelation of F  **actin**  as did intact talin.  Ref: 8586616 J Biochem (Tokyo), 1995
- This work investigates the binding of dystrophin issued from Torpedo marmorata electric organ with  **actin**  in the presence of the phosphoinositide PIP2 that regulates alpha  **actinin**  and filamin binding with  **actin** .  Ref: 7741736 Biochem Biophys Res Commun, 1995
- Several other structural and functional characteristics of fish alpha  **actinin**  were also studied conservation of sequence and domain structure, the role of divalent ions calcium, magnesium and the dielectric constant of the medium in alpha  **actinin**   **actin**  interaction.  Ref: 7584856 Comp Biochem Physiol B Biochem Mol Biol, 1995
- The role of fish alpha  **actinin**  as an anchorage site for thin  **actin**  filaments and elastic titin filaments in Z bands was studied.  Ref: 7584856 Comp Biochem Physiol B Biochem Mol Biol, 1995
- Using antibodies directed against the 27 kDa  **actin**  binding domain of alpha  **actinin** , a reliable carrier for  **actin**  binding sites ABS 1, ABS 2 and ABS 3 also involved in dystrophin and filamin, it was shown that PIP2 affects the ABS 3 environment.  Ref: 7741736 Biochem Biophys Res Commun, 1995
- Cortical  **actin**  and alpha  **actinin**  of the bacterium treated cells showed disorganization, and pericellular fibronectin was degraded by both whole T. denticola and the isolated proteinase.  Ref: 7642269 Infect Immun, 1995
- Fluorescent labeling for  **actin** , talin, alpha  **actinin** , paxillin and phosphotyrosinated components indicated that the organization of all these focal contact associated components was essentially identical in the vinculin containing and vinculin null cells.  Ref: 7673345 J Cell Sci, 1995
- The increase in inositol phosphates is not related to redistribution of cytoskeletal proteins such as  **actin**  or alpha  **actinin**  nor tyrosine phosphorylation of host cell proteins.  Ref: 7607412 FEMS Microbiol Lett, 1995
- The most significant finding was the increase in  **actin**  and alpha  **actinin**  expression in the cytoplasmic compartment after treatment with basic fibroblast growth factor of chick heart cells at Hamburger and Hamilton s stage 19.  Ref: 7675799 Proc Soc Exp Biol Med, 1995
- In  **actin**  bundles, the  **actin**  cross linking protein, alpha  **actinin** , was excluded from sites of myosin localization but concentrated in paired sites flanking each myosin ribbon, suggesting that myosin filament association may initiate a pathway for the formation of  **actin**  filament bundles.  Ref: 7490299 J Cell Biol, 1995
- Hirano bodies are intracellular, paracrystalline, rod like structures which contain  **actin** , tropomyosin, vinculin, alpha  **actinin** , amyloid beta protein precursor and several microtubule associated proteins MAPs.  Ref: 8580423 Neuroreport, 1995
- Aggregation 60 80% of control was restored if 1 unit mL thrombin was added, but there was no corresponding redistribution of  **actin**  binding protein, talin, vinculin, alpha  **actinin** , GPIIb IIIa, PECAM, PI3 kinase, and pp60c src to the cytoskeleton.  Ref: 7626626 Biochemistry, 1995
- To test whether cardiac muscle is a target for regulation by peptide growth factors, we analyzed the effects of two growth factors on  **actin**  and alpha  **actinin**  expression at the subcellular level.  Ref: 7675799 Proc Soc Exp Biol Med, 1995
- These elongated structures contained F  **actin**  and alpha  **actinin**  but were devoid of vinculin.  Ref: 7730414 J Cell Biol, 1995
- Z lines anchor thin filaments from adjacent sarcomeres via the  **actin**  binding function of alpha  **actinin** .  Ref: 7655091 Microsc Res Tech, 1995
- At stage 39, basic fibroblast growth factor induced less marked changes in the accumulation of  **actin**  and alpha  **actinin** .  Ref: 7675799 Proc Soc Exp Biol Med, 1995
- We therefore propose that cadherin catenin complexes are linked to the  **actin**  cytoskeleton via a direct association between alpha  **actinin**  and alpha catenin.  Ref: 7790378 J Cell Biol, 1995
